# Hopping Down the Main Street: Eastern Grey Kangaroos at Home in an Urban Matrix

**DOI:** 10.3390/ani4020272

**Published:** 2014-05-27

**Authors:** Graeme Coulson, Jemma K. Cripps, Michelle E. Wilson

**Affiliations:** 1 Department of Zoology, The University of Melbourne, Parkville, VIC 3010, Australia; 2 Macropus Consulting, 105 Canning Street, Carlton, VIC 3053, Australia; 3 Department of Environment and Primary Industries, Cnr. Midland Highway and Taylor Street, Epsom, VIC 3554, Australia; E-Mail: jemma.cripps@depi.vic.gov.au; 4 Wilson Environmental, 27 Ford Street, Brunswick, VIC 3056, Australia; E-Mail: michelle.wilson@wilsonenvironmental.com.au

**Keywords:** Eastern Grey Kangaroo, citizen science, fecundity, habitat use, matrix-occupying, matrix sensitive, mortality, road-kill, sexual segregation, urban matrix

## Abstract

**Simple Summary:**

Eastern Grey Kangaroos (*Macropus* *giganteus*) occur throughout the seaside town of Anglesea in southern Victoria, Australia. We have tagged about half of these kangaroos in a longitudinal study of population dynamics and behavior. A golf course forms the nucleus of this population. Females live on and around the golf course, but males roam across the town in autumn and winter, living in bush reserves, empty blocks and back yards. Most females breed every year, but over half of their young disappear. Vehicles are the major cause of adult deaths, killing a much higher proportion of males than females.

**Abstract:**

Most urban mammals are small. However, one of the largest marsupials, the Eastern Grey Kangaroo *Macropus* *giganteus*, occurs in some urban areas. In 2007, we embarked on a longitudinal study of this species in the seaside town of Anglesea in southern Victoria, Australia. We have captured and tagged 360 individuals to date, fitting each adult with a collar displaying its name. We have monitored survival, reproduction and movements by resighting, recapture and radio-tracking, augmented by citizen science reports of collared individuals. Kangaroos occurred throughout the town, but the golf course formed the nucleus of this urban population. The course supported a high density of kangaroos (2–5/ha), and approximately half of them were tagged. Total counts of kangaroos on the golf course were highest in summer, at the peak of the mating season, and lowest in winter, when many males but not females left the course. Almost all tagged adult females were sedentary, using only part of the golf course and adjacent native vegetation and residential blocks. In contrast, during the non-mating season (autumn and winter), many tagged adult males ranged widely across the town in a mix of native vegetation remnants, recreation reserves, vacant blocks, commercial properties and residential gardens. Annual fecundity of tagged females was generally high (≥70%), but survival of tagged juveniles was low (54%). We could not determine the cause of death of most juveniles. Vehicles were the major (47%) cause of mortality of tagged adults. Road-kills were concentrated (74%) in autumn and winter, and were heavily male biased: half of all tagged males died on roads compared with only 20% of tagged females. We predict that this novel and potent mortality factor will have profound, long-term impacts on the demography and behavior of the urban kangaroo population at Anglesea.

## 1. Introduction

Urbanization replaces natural environments with two novel habitat types: ‘grey spaces’, where >80% of the area is covered with buildings and hard surfaces, and ‘green spaces’, which include managed vegetation (e.g., gardens and golf courses), as well as patches of unmanaged and remnant vegetation [1]. The resulting urban matrix is characterized by different ecological processes compared with non-urban environments [2,3]. Wildlife can be classified according to their sensitivity to the grey and green components of the urban matrix: matrix-occupying, matrix-sensitive or urban-sensitive [4]. Matrix-occupying species, such as the House Mouse *Mus musculus* in England [5] and the Blackbird *Turdus merula* and Magpie *Pica* *pica* in Europe [6], typically dominate the urban matrix due to their ability to move through and live within the grey spaces. Matrix-sensitive species, such as the American Crow *Corvus brachyrhynchos* in the USA [7] and the European Hedgehog *Erinaceus europaeus* in England [8], perceive the grey spaces as unsuitable habitat, lacking food and shelter resources and forming a barrier to movement; these species are usually restricted to green spaces within the urban matrix, where patches of vegetation provide the only suitable habitat. The third group, urban-sensitive species such as the Small Vesper Mouse *Calomys laucha* and Azara’s Grass Mouse *Akodon azarae* in Argentina [9] and the Growling Grass Frog *Litoria raniformis* in Australia [10], are unable to persist within the grey-green urban matrix, and are often threatened by urbanization.

Most research on urban wildlife has concerned birds and their use of the habitat matrix [11], but mammals have also received some attention. Baker and Harris [12] pointed out that urban mammals show a strong effect of body size on population viability: the majority of matrix-occupying and matrix-sensitive species weigh less than 10 kg, their small size allowing them to exploit a wide range of food in small habitat patches as they move easily and unobtrusively throughout the grey-green matrix. North American deer, however, are a clear exception to this pattern. White-tailed Deer *Odocoileus virginianus* have established robust populations in the urban matrix in many areas of the USA [13,14,15,16,17] and in Canada [18]. Its sister species, the Mule or Black-tailed Deer *Odocoileus hemionus*, has penetrated the urban matrix to a lesser extent, but has established urban populations in some areas [19,20]. The dominant management issue presented by these urban deer is the high incidence of collisions with vehicles, and their associated impacts on animal welfare, human health and property damage [16,18,21]. Furthermore, urban deer in North America often host the tick *Ixodes* *scapularis*, the vector of the causal agent (*Borrelia burgdorferi*) of Lyme disease [22]; the proximity of deer increases exposure to ticks, facilitating the transmission of this debilitating and sometimes fatal zoonotic disease in humans.

Macropodid marsupials (kangaroos and wallabies) are ecological analogues of deer [23], so might also be expected to occupy urban areas. Despite the common misconception that kangaroos routinely hop down the main streets of many Australian towns [24], very few urban populations have been documented. Like North American deer, the few reported cases are exceptions to the rule that most urban mammals are small (<10 kg) [12]. With females weighing ≤15 kg and males ≤21 kg [25], the Swamp Wallaby *Wallabia bicolor* occupies peri-urban patches of native vegetation around Sydney, New South Wales. [26,27]. The Agile Wallaby *Macropus agilis*, similar in size to the Swamp Wallaby [28], has also established a population in a peri-urban reserve in Darwin, Northern Territory [29]. The Western Grey Kangaroo *Macropus* *fuliginosus* is larger, with females weighing ≤39 kg and males ≤72 kg [30]; this species occurs on some golf courses in the suburbs of Perth, Western Australia [31]. However, all three species appear to be matrix-sensitive, since they use only the green spaces in their urban matrices. In contrast, the Eastern Grey Kangaroo *Macropus* *giganteus* is arguably the most urban of the macropodids, Although it is larger than the other species, with females weighing ≤42 kg and males ≤85 kg [32], it is apparently a matrix-occupying species in some parts of its range, using both grey and green spaces within the matrix. The best-known population of Eastern Grey Kangaroos lives within and on the fringe of the city of Canberra, Australian Capital Territory, which is known as ‘the bush capital’ [33]. Ballard [34] also described a population living amongst a retirement community in Port Macquarie on the north coast of New South Wales, and Inwood *et al.* [35] reported another urban population in Anglesea on the west coast of Victoria. Social surveys at these three sites revealed that collisions with vehicles were the dominant management issue, as for North American deer, but people were also concerned about the risk of attacks by kangaroos [33,34,35].

The urban kangaroo population at Anglesea is the subject of a community-based management program, developed in response to the issues raised by the human residents [35]. Three key issues that emerged were: (1) improved understanding of the biology of Anglesea’s kangaroos, particularly their health, demographics and movements; (2) monitoring biological change over time, and assessing the outcomes of management actions; (3) mitigation of kangaroo-vehicle collisions by identifying hotspots. In 2007 we began a program of applied research aimed at addressing these issues. We adopted a longitudinal approach, capturing and marking a large sample of the population [36], then monitoring these individuals through time. Studies such as these have proven valuable in a variety of wildlife species [37], but a long-term project on marked kangaroos had not previously been undertaken. Crucially for our project, we named each individual and displayed its name on its collar, then used conventional monitoring techniques augmented by citizen science reports to monitor individuals [38]. This marked population formed the foundation for ancillary studies of the efficacy of anthelmintic drugs [39] and contraceptive implants [40], and the effect of reproduction on feeding behavior [41], which have contributed to the overall research program. We have also coupled the Anglesea program with a second longitudinal study, commenced in 2008, of a more natural population at Wilsons Promontory National Park, southern Victoria. Projects are underway at both sites on factors influencing the age at maturity, timing of births, sex ratio of young, and reproductive success of both sexes. This paper reports on our first six years of urban kangaroo research at Anglesea, and assesses our progress towards the three key issues of concern to Anglesea residents.

## 2. Methods

**Figure 1:**
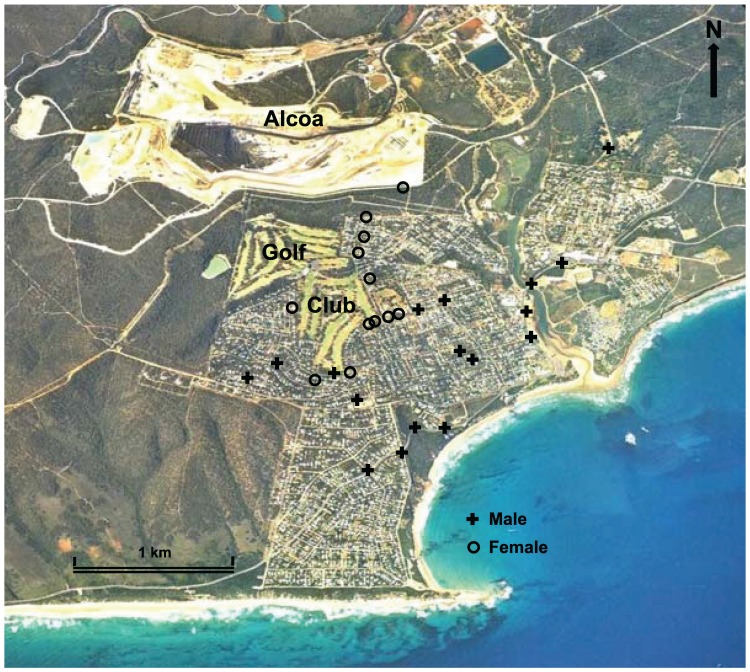
Aerial photograph of Anglesea, Victoria, Australia, showing the compact residential area bounded by the Anglesea Heath, the Anglesea Golf Club (Golf) within the town boundary, and the Alcoa Australia coal mine and power station (Alcoa) to the north of the town. Also shows locations of road-kills of tagged male and female Eastern Grey Kangaroos *Macropus giganteus* reported from 2008 to 2013.

Anglesea (38°40'S, 144°19'E) is located on the Surf Coast in southern Victoria, Australia (Figure 1). The town has about 2000 permanent human residents; the population increases at weekends and rises dramatically during summer, exceeding 10,000 at peak times [42]. Anglesea lies on the iconic Great Ocean Road, a major attraction for domestic and international tourists, and many include kangaroo viewing in their itinerary. Kangaroo imagery promotes the town: banners in the main street declare Anglesea to be ‘where bush meets sea’, the Anglesea Primary School boasts a ‘kangaroo on a surfboard’ logo, and the Anglesea Golf Club features a kangaroo on its flag and signage around the course. The town has a riverside park as well as a number of small reserves of remnant native vegetation within the urban matrix. The Surf Coast Shire, the local government authority, imposes strict regulations against clearing of vegetation on private land, and many residential blocks are well vegetated. Many blocks are unfenced and some streets are unsealed. The urban area lies within the Anglesea Heath; this forms part of the Great Otway National Park, which is an extensive, continuous reserve of native vegetation along much of the Great Ocean Road. Anglesea Heath is jointly managed by Parks Victoria and Alcoa Australia, which operates an open-cast brown coal mine and coal-fired power station in the heath north of the town [43].

### 2.1. Study Sites

**Figure 2:**
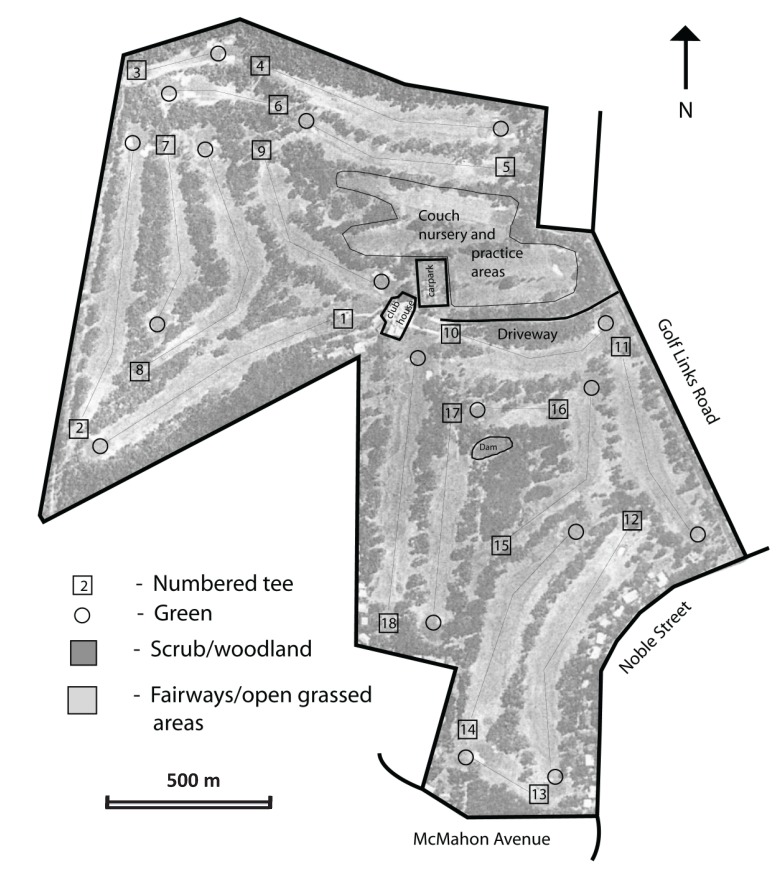
Aerial photograph of the Anglesea Golf Club, Victoria, Australia, showing the layout of the 18-hole course and the contrast between open foraging areas and vegetated shelter for Eastern Grey Kangaroos *Macropus giganteus*.

The Anglesea Golf Club has an 18-hole course of tree-lined fairways (Figure 2) planted with Couch Grass *Cynodon dactylon*, which is irrigated and fertilized, as are the tees and greens. The Front Nine (holes 1–9) is 32 ha in area, including practice areas and a Couch Grass nursery. The Front Nine is bounded to the north and west by the Anglesea Heath, an extensive area of native vegetation dominated by eucalypts with an understory of low shrubs. Its southern side is bounded partly by a band of remnant native woodland bordering residential streets, and by the entrance road to the clubhouse; the eastern side is bounded by Golf Links Road, with houses on the opposite side. The Front Nine is unfenced, so kangaroos can move freely in all directions across these boundaries. The Back Nine (holes 10–18) is 27 ha in area. It is bounded to the south and west by houses facing away from the course; many are unfenced so kangaroos can move through the yards onto residential streets. Its northern and western sides are demarcated by a 1.8-m high steel mesh fence, which is designed to stop kangaroos moving onto Golf Links Road and the entrance road, and to restrict access to the course by tourists. Each half of the course has a 2-ha patch of remnant native vegetation, roughly in the center, providing additional refuge for the kangaroos.

Camp Wilkin is a 6-ha school camp at the junction of Golf Links Road and Noble Street, located on the opposite side of the fence road bordering the Back Nine of the golf course. The camp consists of a building complex (bunkrooms, dining room, gymnasium, and staff housing) at the corner of two residential streets, as well as a sports field, adventure equipment and remnant native vegetation at the rear of the camp, where kangaroos shelter during the day. The perimeter of the camp has a tall paling fence on two sides, which effectively contains kangaroos, and a low wire fence on the other two sides, which they can hop over. However, most kangaroo movement occurs onto the streets through vehicle gates.

### 2.2. Capture and Marking

To capture kangaroos we exploited their habituation to people on the golf course and at the school camp, using two techniques that have proven to be safe and selective. For captures at close range we used a 10-mL Paxarms syringe attached to a light-weight telescopic pole [36]. The smallest of these was a two-piece aluminum pole, which extended to 1.4 m and superficially resembled a golf club. For less approachable individuals we used longer (≤3.6 m) two-piece poles, or even longer (≤5.3 m) three-piece poles if necessary. We approached kangaroos slowly and injected Zoletil 100 (1:1 Zolezapam and Tiletamine) into the muscle mass of the hind-limb at a dose rate of approximately 5 mg/kg. Adult kangaroos that were newly arrived at the golf course were often too wary to capture with a pole syringe, as were some individuals that had been captured repeatedly. We captured these individuals using a WildVet Para-medic band-powered bow (range ≤ 8 m) or a WildVet Pro-medic crossbow (range ≤ 20 m). Both bows fired lightweight injection arrows into the hind-limb muscle with minimum impact trauma, and injected a dose of approximately 5 mg/kg of Zoletil 100.

To date we have captured and marked 360 individual kangaroos: 211 (68 male, 143 female) as adults, 15 (8 male, 7 female) as sub-adults, and 39 as young-at-foot (22 male, 17 female) closely associated with their mothers after pouch exit. In addition, 83 young in the pouch (55 male, 37 female) were large enough to tag when we captured their mothers. We have recaptured adults on 269 occasions, comprising one (57% of adult recaptures), two (28%), three (10%), four (4%) or five (1%) recaptures per individual. We have also recaptured 40 sub-adults up to three times each, and 39 young-at-foot once or twice each. All but 17 of our captures of adults, sub-adults and young-at-foot were on the golf course; we captured most (16) of the others at Camp Wilkin, plus one in a small reserve beside the Anglesea River. We used the small pole for the majority (56%) of captures, two medium poles for 20% of captures and two long poles for 19% of captures. For the remainder of captures of more wary kangaroos we used either the Para-medic bow (4%) of the more powerful Pro-medic bow (2%).

Once a kangaroo had been injected by pole syringe or arrow, we withdrew a short distance and kept the kangaroo under observation to guard against approaches by people, dogs, cars or aggressive conspecifics, and intervened when necessary on occasions. Induction was rapid (about 5 min), resulting in immobilisation and anaesthesia for approximately 1 h. When the kangaroo was immobilised, we transferred it to a shade-cloth cradle to weigh it, and took a standard set of body measurements. We marked each adult and sub-adult with a unique combination of two or three colored Allflex ear-tags, with an individual identity number and enhanced by color-matched 3M reflective tape for identification at night. If pouch young were furred and had erect ears, we marked them with a unique color combination of two Leader swivel tags, which we also numbered and matched with reflective tape. We followed the same procedure for a few young-at-foot, which we captured soon after they had left the pouch permanently. Insertion of the Allflex and Leader tags displaced a small piece of ear tissue, which we collected for genetic analysis. We fitted each adult male with a 45-mm wide collar made of Ritchey trilaminar plastic, overlapped and fastened with two steel nuts and bolts; this design proved moderately resistant to damage incurred during fights between males. We fitted some adult females with 35-mm wide collars of this material, but gave most a softer and more flexible 35-mm wide collar made of a doubled strip of Innova International UV-stable vinyl, overlapped and fastened with four pairs of ITW Fastex ratchet rivets. We gave each individual a unique three, four or sometimes five-letter name (e.g., *Nat* and *Vlad*), and wrote the name in large letters with a black Allflex tag pen two or three times around the collar. After examination and marking, we moved each kangaroo to a quiet, sheltered area and allowed it to recover without further disturbance.

### 2.3. Population Surveys

In 2010 we began twice-yearly surveys of the golf course population, one in winter and another in late summer. Two of us (GC and JC) conducted total counts of kangaroos in the morning, starting at the beginning of civil twilight, and in the evening, starting 1.5 h before the end of civil twilight, when most kangaroos were feeding in the open on the fairways. We modified the procedure used by Inwood *et al.* [33]: instead of walking along all the fairways in the order of play, we walked steadily along the trees lining the fairways so that we could effectively scan pairs of fairways, thus minimizing potential double-counting of kangaroos that moved between fairways and allowing us to cover the course more rapidly at the optimal time of day. We used binoculars to scan groups of kangaroos from distances ≤50 m, counting all adults, sub-adults and young-at-foot, but excluding young that temporarily emerged from the pouch. From the start of 2011 we also recorded each individual as marked (with ear-tags and/or collar) or not. To have two independent counts for each side, we each started on opposite sides of the course, then swapped to the alternate side. We discounted any surveys where an obvious disturbance (e.g., by grounds staff or dogs) might have caused a double count. We took the higher of our two counts on each side as the total for each morning or evening session, and took the highest session total as the minimum population size for each survey period.

At least once each season we conducted a census of the kangaroo population at the Golf Club and at Camp Wilkin, augmented by occasional sightings away from these focal areas (see Section 2.4. Movements). We recorded the presence of each marked individual and its location on the golf course or school camp. We also recorded the reproductive status of each female, any loss of tags or collars, and any obvious injuries or marked changes in body condition or behavior.

### 2.4. Movements

We obtained data on the movements of marked kangaroos beyond the golf course from five sources: citizen science reports, street searches, camera traps, road-kills and radio-tracking. Naming the kangaroos and displaying the names on their collars proved to be a key element in engaging public interest in our research program [38]. To encourage citizen science reports of marked kangaroos around the town, we gave talks to a range of community groups and to all year levels at the local primary school, and spoke about the program at other events during the year. We also put up posters in shop windows and community noticeboards, and distributed contact cards to local organizations and interested individuals. In addition, our research program was covered by television documentaries, local newspapers and community newsletters.

We conducted searches of the streets around the town from a vehicle, aided by a spotlight at night. With experience, we were able to identify a number of locations where kangaroos could usually be seen, and returned to them often. Road-kills of marked kangaroos were reported by members of the public, wildlife carers and local police, who were often called out to shoot badly-injured kangaroos [35].

In the autumn of 2012 we selected five marked adult males that were often not present on the golf course, with the goal of qualitatively assessing the types of habitats occupied. We recaptured them and fitted them with a reflective Sirtrack radio-collar. The collars weighed <250 g, incorporated a mortality switch, and transmitted in the 150–151 MHz band with a range of approximately 1.5 km. In the following winter we tracked them at irregular intervals at night and during the day. We located each individual off the golf course two to seven times, initially by triangulation from vantage points or by driving around the town with an omnidirectional antenna on the roof of the vehicle, then by homing in on foot. Once we had located them, we recorded the habitat they occupied and the identity of any other marked kangaroos nearby.

### 2.5. Mortality

We found some carcasses of marked kangaroos on the golf course and nearby streets. However, most deaths of marked kangaroos were reported to us by Golf Club staff, police, wildlife carers and concerned residents. When a carcass was found, we confirmed the kangaroo’s identity from its ear-tags and/or collar, recorded its location, and determined its cause of death when possible. We also attempted to retrieve its body and take the same morphometric measures recorded in life, except where the body parts were damaged, then removed the head and allowed it to decompose so that we could later age the clean skull by molar index [44]. In three cases when the carcass was fresh and the cause of death was unclear, we submitted the kangaroo for formal post-mortem examination.

## 3. Results

### 3.1. Abundance and Attendance

**Figure 3:**
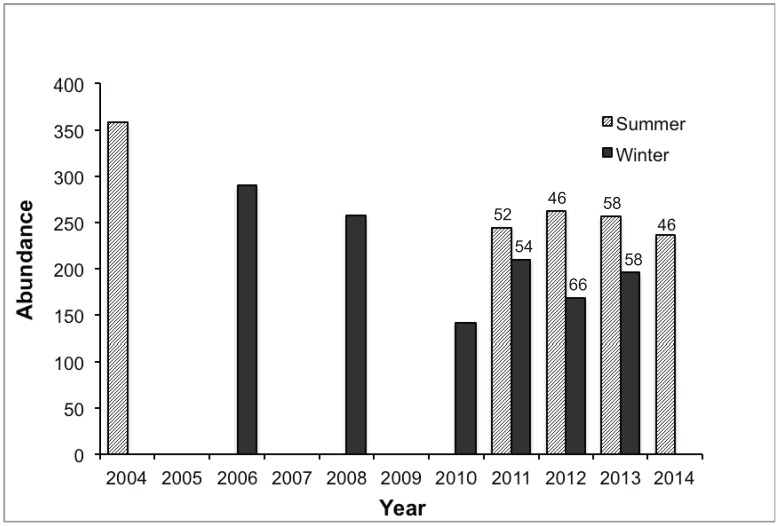
Abundance of Eastern Grey Kangaroos *Macropus giganteus* at Anglesea Golf Club from surveys conducted in winter and summer, inconsistently at first, from 2004 to 2014. Data for 2004 and 2006 compiled from Inwood *et al.* [35].

The abundance of kangaroos on the golf course declined by about 100 animals in the last decade, from a peak of 359 in summer 2004 to 237–263 in the last four summers (Figure 3). Winter counts have shown a similar decline, from 290 in 2006 to 142–210 in the last four winters (Figure 3). An oscillating pattern of winter troughs and summer peaks in abundance has also been evident since twice-yearly surveys began in 2010. In the last seven surveys, 46–66% of the kangaroos seen were tagged (Figure 3).

**Figure 4:**
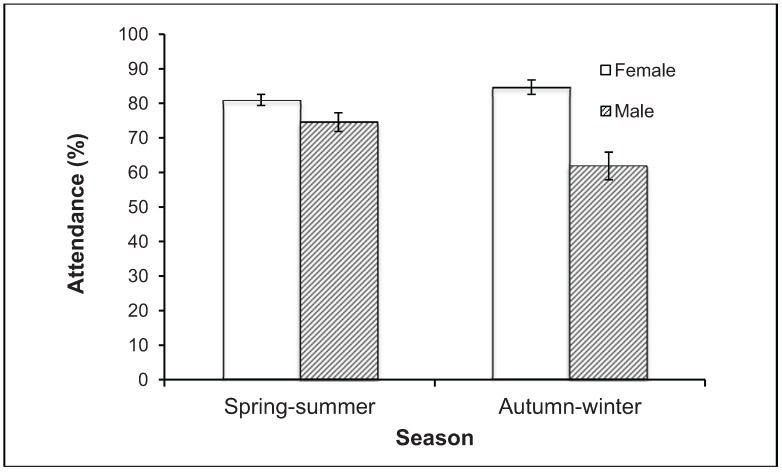
Mean attendance rate of individual male and female Eastern Grey Kangaroos *Macropus giganteus* (tagged as adults) in autumn/winter and spring/summer on the Anglesea golf course from May 2008 to November 2013. Data from censuses recording ≥50 tagged adults (8 autumn/winter and 16 spring/summer), from the first census after each individual was tagged to the last in which it was known to be alive, excluding individuals spanning <10 censuses. Error bars show standard error.

To assess the attendance of tagged kangaroos at the golf course, we scored individuals as present or absent in each census from May 2008 to November 2013. We included only censuses that recorded ≥50 tagged adults to ensure an adequate sample size. This gave eight censuses in autumn and winter (March to August), two in each year of the four years, and 16 censuses in spring and summer (September to February), spread almost evenly over the years. We scored only individuals tagged as adults (76 females and 34 males), from the first census after each individual was tagged to the last census in which it was known to be alive, excluding individuals that spanned <10 censuses to ensure adequate coverage of the two ‘seasons’. The overall attendance rate was high, with a mean (±SE) of 79 ± 1% (Figure 4). Attendance was not affected by season (Repeated-measures ANOVA, F = 0.51, df = 1,108, P = 0.478), but the attendance rate of females was higher than males (F = 25.25 df = 1,108, P < 0.0001). Season and sex also interacted (F = 15.48, df = 1,108, P = 0.0001): attendance by males was much lower in autumn-winter (62%) than in spring-summer (75%), whereas females remained above 80%.

### 3.2. Fecundity and Mortality

We recorded the number of reproductive attempts by each tagged female based mainly on a visible bulge indicative of a young in the pouch. In some cases, pouch young were too small to detect externally but were recorded when we captured the female. The overall level of fecundity of adult females was low in the first two full years of this study: 39% in 2008 (n = 77) and 38% in 2009 (n = 65). Fecundity in the following four years was consistently higher: 70% in 2010 (n = 96), 76% in 2011 (n = 91), 74% in 2012 (n = 78) and 75% in 2013 (n = 63). These values excluded any of the females treated with fertility control implants by Wilson *et al.* [40], but included females used as procedural (untreated) controls in that study.

A high proportion (46%) of the 221 juveniles that we tagged subsequently disappeared from the population before the age of three years. Although we rarely found their carcasses, we concluded that these juveniles had died, because they were still dependent on, or closely associated with, their mothers at the time. Of these juveniles, 41% disappeared in their first year, mostly towards the end of the ten-month pouch life, 54% in their second year, and the remainder (4%) in their third year.

Adult mortality could not be distinguished from dispersal from the golf course or school camp, so we did not estimate the mortality rate of adults. However, we were able to confirm the deaths of 78 tagged adults. More than half (55%) of these were found dead on the golf course, or on streets or in reserves elsewhere in the town. The other cases involved seriously injured or moribund kangaroos on the golf course or around the town, which were either shot by police (31%) or given lethal injections by wildlife rescuers (14%). The sex ratio (1 male:1.53 females) of recorded deaths did not differ from the ratio of adults originally tagged (Contingency Chi square, χ^2^ = 2.86, df = 1, P = 0.091. The estimated age of the skulls of adults that we retrieved (n = 23) ranged from 2.8 to 18.7 (median = 8.9) years. We were unable to determine the direct cause of 53% of adult deaths. One died when struck by a golf ball, five sustained traumatic injuries from unknown sources, and four were probably attacked by Dogs or European Foxes *Vulpes vulpes*, which were present on the golf course at the time.

Collisions with vehicles were the major (47%) known cause of death. These road-kills had a strong seasonal bias: of 37 incidents that could be assigned to a season, 32% occurred in autumn and 41% in winter, whereas only 14% occurred in each of spring and summer (Goodness-of-fit G-test, G = 42.85, df = 3, P < 0.0001). However, there was no difference in the frequency on weekdays *versus* weekends for the 29 incidents assigned to a specific day (Goodness-of-fit G-test, G = 0.29, df = 1, P = 0.594). Unlike overall deaths, collisions were highly sex biased: exactly half of all males originally tagged as adult died on roads, *versus* 20% of adult females tagged (Contingency Chi-square, χ^2^ = 20.55, df = 1, P < 0.0001). The spatial distribution of road-kills also differed markedly between the sexes: with the exception of one female (*Grace*) on the edge of town and 0.5 km from the golf course, all road-kills of females were on streets within a block of the golf course, whereas males were killed throughout the town (Figure 1).

### 3.3. Movement and Habitats

**Figure 5:**
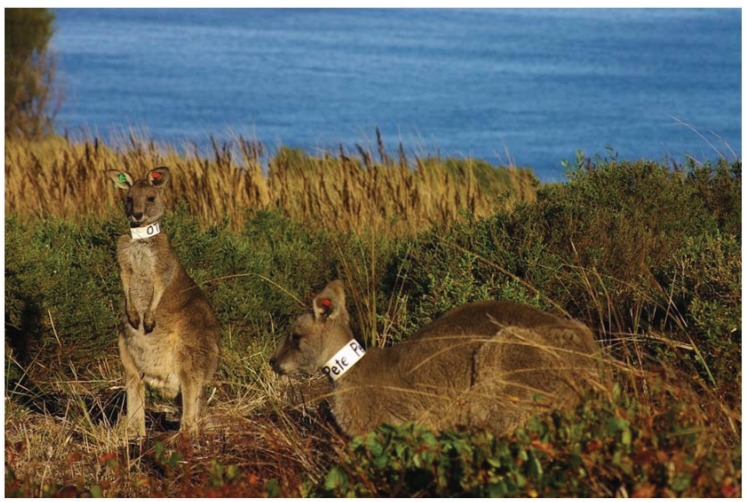
Two tagged and collared male Eastern Grey Kangaroos *Macropus giganteus* (*Otis* and *Pete*) at the Anglesea Lookout Flora Reserve above the Great Ocean Road, about 1 km south of where they were collared and tagged at the Anglesea Golf Club.

Tagged kangaroos moved between the golf course and Camp Wilkin. Of the ten individuals first captured at Camp Wilkin, we resighted five (all female) only at the camp, but saw the others (three male, two female) on the golf course on occasions. Another seven individuals (four male, two female) that had first been captured on the golf course were seen at Camp Wilkin. We also received citizen science reports of tagged kangaroos, primarily males, throughout the town, and augmented these with our own observations (Figure 5). In addition, we located radio-collared males in many parts of the town, and radio-tracking one individual often led us to other tagged males nearby. These movements off the golf course and into the town matrix occurred mainly in autumn and winter. Males sometimes also left the golf course in summer. For example, one large male (*Stan*) was reported resting during the day and drinking from a pond in a shady backyard on hot (>40 °C) days in two consecutive summers. However, we had insufficient locations of tagged individuals for quantitative analyses of habitat use. At a qualitative level, kangaroos occurred in a wide range of habitats beyond the golf course and school camp: bush reserves, public playgrounds, holiday parks, vacant blocks, commercial properties and residential gardens. We also located some kangaroos in native vegetation of the Anglesea Heath on the fringe of the town.

We detected only four tagged kangaroos moving beyond the town boundary. One young adult male (*Max*) was killed by a car on the Great Ocean Road, 3.4 km northeast of his last sighting on the golf course. A second male (*Ben*) was reported a number of times in the neighboring town of Aireys Inlet, 6.6 km southwest of the golf course, and was ultimately found dead 12 months after initial reports, on the Great Ocean Road on the edge of Aireys Inlet. One young female (*Boo*) was detected on a camera trap set for a fauna survey in the Anglesea Heath, 8.6 km north-northwest of the golf course and three years after we last saw her there. A second adult female (*Vahn*) was detected on another camera trap set to monitor European Foxes in the Anglesea Heath, 7.7 km west of the golf course, and 4 months after her last sighting on the course.

## 4. Discussion

Many wildlife species exhibit adaptations to the novel and often-stressful conditions they experience in urban environments [6,45]. For example, long-term studies of Florida Key Deer *Odocoileus virginianus* *clavium* show that they have adapted to urbanization by increasing their use of urban habitat, and their body weight and survival is now higher than in less urban deer [15]. However, long-term studies of urban wildlife are extremely rare in Australia [4]. As the first long-term study of tagged Eastern Grey Kangaroos, our set of findings cannot yet be contrasted with the demography and behavior of other kangaroo populations in more natural settings. Our study also has many years to run, and we cannot yet determine the extent of demographic or behavioral change within the kangaroo population at Anglesea. Nonetheless, we can explore some elements of kangaroo ecology at Anglesea and identify potential effects of urbanization.

### 4.1. Population Dynamics

The density of the kangaroo population on the 73-ha golf course has ranged from 4.9/ha at its peak in summer 2003–04 to 2.0/ha in winter 2010, when we began twice-yearly surveys. The density in summer has since ranged from 3.3/ha to 3.6/ha. These densities were equal to or higher than any in the peri-urban reserves reviewed by Adderton Herbert [46], and are similar to the maximum densities recorded in the Australian Capital Territory [33]. These comparisons suggest that the population density of kangaroos can be enhanced by the urban environment, as it has been for many wildlife species elsewhere in the world [6,15].

The decline in abundance since the peak in summer 2003–04 has no obvious cause. A sample of 42 adult female kangaroos was treated with contraceptive implants in 2008 as part of an ancillary study of fertility control techniques [40,41]. The efficacy of one compound (deslorelin) was limited to one or two years, whereas the other (levonorgestrel) has continued to be effective [47]. However, this experiment also included 23 control (untreated) females, and other females, both tagged and untagged, were not treated with contraceptives. It is unlikely that fertility control caused the population to decline, because to achieve population stability in a long-lived, annually-breeding species like the Eastern Grey Kangaroo, it is necessary to contracept a high proportion (>90%) of a population [48]. A more likely explanation for the decline is that the kangaroo population at Anglesea was under pressure from the ‘millennium drought’, a long-term rainfall deficit across south-eastern Australia from 2001 to 2009 [49]. While the permanent water and irrigated fairways on the golf course would have buffered the kangaroo population against drought, the course may not have been able to support densities approaching 5/ha indefinitely. Anecdotal observations of kangaroos feeding on roadside verges, and an increase in fatal incidents reported by police over a decade [35], support this interpretation. The unusually low fecundity (<40%) of adult females in 2008 and 2009 was also consistent with the impact of drought in other populations of Eastern Grey Kangaroos [50].

A pattern of summer peaks and winter troughs in abundance has been evident since 2010. Breeding is seasonal at Anglesea [40], with a peak of births in early summer generating a pulse in recruitment as young leave the pouch permanently the following spring. However, young tagged in the pouch disappeared at a high rate (46%), and 41% of those were in their first year, so any recruitment pulse would likely have been muted by the time we conducted population surveys later in summer. Banks [51] reported a similar rate of loss of juvenile Eastern Grey Kangaroos, which he attributed to predation by European foxes. The subsequent trough in winter would reflect a combination of continued disappearance (54%) of juveniles in their second year, coupled with the lower attendance rate (61%) of adult males on the golf course.

### 4.2. Movement

We detected very few large-scale movements of tagged kangaroos. However, our reliance on citizen science reports, augmented by our own sightings, undoubtedly generated a detectability bias. While the dispersal and subsequent road-kills of two young males (*Max* and *Ben*) were reported by citizen scientists, the long-range movements into the Anglesea Heath by two adult females (*Boo* and *Vahn*) would have gone undetected without camera traps set for other purposes. However, other movement studies of Eastern Grey Kangaroos, based either on sightings of tagged individuals [52] or radio-tracking [53,54,55], have also shown that they are largely sedentary. We are unable to assess whether the home range size of kangaroos in the urban matrix of Anglesea is smaller than in more natural landscapes, as has been reported for well-studied white-tailed deer [15,56,57].

Adult males exhibited strong sexual segregation in space, as reported for this species in natural landscapes [58]. The attendance of females on the golf course was high throughout the year, whereas attendance of males declined in the non-breeding season (autumn and winter). Males were then more likely to be encountered throughout the town in a variety of habitats, and road-kills of males were widespread across the town, unlike those of females, which were almost all near the golf course. The incidence of road-kills was considerably higher in the non-breeding season, when 73% of deaths occurred. The incidence of road-kills was also heavily biased towards males. Similar male bias in the frequency of road-kills has been reported for Eastern Grey Kangaroos in the peri-urban landscape around Canberra [59] and in rural landscapes [60,61]. However, these studies lacked data on the sex composition of the populations from which the road-kills were drawn, so could not assess the impact of vehicles as a sex-biased mortality factor. At Anglesea, the impact was clearly male biased: half of the males that we tagged were definitely killed on roads, whereas only one fifth of tagged females were.

## 5. Conclusions

The Eastern Grey Kangaroo has a broad geographic distribution, and occurs in a wide variety of habitats, ranging from native forests, woodlands (including mallee scrub), shrublands and heathland, to modified habitats such as pine plantations and golf courses [32]. These habitats all provide a mix open grassy foraging areas and cover from the weather and predators [32]. At a landscape scale, the density of this species is highest when these dual resources occur in a mosaic, so that forage and cover are in close proximity [62]. Our study at Anglesea suggests that the urban matrix also provides a suitable mosaic of forage and cover, and that kangaroos are able to move through and live within the matrix at the scale of a residential block. Therefore the kangaroos of Anglesea can be classified as matrix-occupying [4] and, like North American deer, do not conform to predictions based on body size [15]. However, our study shows that the golf course forms the nucleus of this population. The nearby school camp (Camp Wilkin) supports a satellite sub-population, exchanging individuals with the golf course, but we are confident that there is no other source of kangaroos anywhere in the town.

Although Eastern Grey Kangaroos occupied the urban matrix in Anglesea, strong sex differences were evident. Females exploited the mix of forage and cover provided by the golf course and adjacent residential blocks; males were less reliant on the golf course and moved further away during autumn and winter, occupying small patches of forage and cover throughout the town. This disparity suggests that, like urban Coyotes *Canis latrans* in North America [63], kangaroos have retained their mating system in this urban matrix. Kangaroos are polygynous and show sexual segregation during the non-mating season [58]. In this urban environment, the segregative behavior of males exposes them to greater risk of road-kill, and probably to other disturbance from people and Dogs. This novel and potent mortality factor, which operates selectively against males, is likely to have profound impacts on the demography and behavioral ecology of urban kangaroos in the future.

The aim of this study was to address the three key kangaroo issues raised by the human residents of Anglesea. The first of these issues was a desire to understand more of the biology of Anglesea’s kangaroos, particularly their health, demographics and movements. Animal health remains poorly understood, although an ancillary study [64] has examined the influence of gastrointestinal parasites on growth, body condition and haematological values of juvenile kangaroos at Anglesea. We now have a more complete understanding of the demography of this population, notably the generally high rate of female fecundity, which is largely offset by high juvenile mortality. Nevertheless, we have been unable to elucidate the causes of mortality in roughly half of adults and juveniles. Our sightings and citizen science reports of tagged kangaroos have revealed strong sex differences in ranging behavior, and radio-tracking a small sample of adult males has further illuminated their use of the urban matrix. However, we frequently encountered logistical challenges posed by radio interference and trespass onto private property, which are typical of radio-tracking in urban environments [65,66], so we have begun a pilot study of Global Positioning System collars (fitted to one male and one female), designed to log locations at 30-min intervals for 18 months. The second issue was the need to monitor biological change over time, and assess the outcomes of management actions. Our twice-yearly surveys of the kangaroo population on the golf course have shown a long-term decline in abundance, although its underlying causes are obscure, and strong seasonal oscillation in use of the course by adult males. No specific management actions have been undertaken since we began this study, but we now have a solid information base to assess management outcomes. The final management issue was mitigation of kangaroo-vehicle collisions by identifying hotspots. Road-kills have proven to be a dominant mortality factor, and several hotspots can now be identified. More importantly, our finding of strong spatial segregation by adult males in the autumn and winter non-breeding season has clear implications for the human residents: collisions with large male kangaroos can occur throughout the town on any day in half of the year. This point will become a key message in learning to live with kangaroos in Anglesea.
